# Neonatal hyperbilirubinemia

**DOI:** 10.1186/1824-7288-40-S2-A10

**Published:** 2014-10-09

**Authors:** Matteo Dal Ben, Silvia Gazzin, Claudio Tiribelli

**Affiliations:** 1 Centro Studi Fegato, Fondazione Italiana Fegato, AREA Science Park, Campus Basovizza, 34149 Trieste, Italy

## 

Unconjugated hyperbilirubinemia is a common condition in the first week of postnatal life. Low levels of bilirubin exert antioxidant effects, but some neonates may develop very high levels of unconjugated bilirubin (UCB), with an increase of the unbound free fraction (B_f_), able to diffuse through the blood brain barrier.

Amount and duration of hyperbilirubinemia and the neurodevelopmental age (preterm neonates) at the time of insult exposition is supposed to influence the location of selective brain damage, as well as the severity of consequences [1]. The clinical manifestations range from the less severe Bilirubin-Induced Neurological Damage (BIND) to a more severe chronic kernicterus, while the regional selectivity of damage may elicit to motor disorders and athetosis (basal ganglia and cerebellum), auditory dysfunction (inferior colliculus), memory and learning impairment (hippocampus) [2].

To avoid neurological consequences, the Clinical Practice Guideline of the American Academy of Pediatrics recommend total bilirubin determination (TSB) on every jaundice infant, both during hospital stay and post discharge follow-up. To this goal, Bilistik [3], a new minimally invasive method for measuring TSB, may improve substantially the triage of jaundice newborns with potential risk of brain damage and kernicterus to be addressed to phototherapy. Moreover, recent studies have raised concerns about the potential toxicity of intensive phototherapy in preterm neonates, and no information about its effectiveness in quickly reducing brain bilirubin concentration are yet available [4].

Early neuronal accumulation of bilirubin in damaged regions and its brain metabolism may have a role in the marked regional differences observed in kernicterus impairment. This hypothesis is supported by the role of brain cytochrome P-450 (Cyp), known to oxidize UCB. In the brain of Gunn rats, an early upregulation of Cyp mRNAs was observed in the unaffected brain regions, cortex and superior colliculus, in contrast to the delayed and slight upregulation observed in the affected regions, inferior colliculus and cerebellum [6], where UCB alters the cell cycle inducing apoptosis [7].

Clarification of pathophysiology of UCB neurotoxicity, that continues to be an important risk among newborns worldwide, may open new perspectives for therapeutic approaches, focused in protecting directly the brain, the final target of bilirubin toxicity. To this aim the development of new research models, appear of particular relevance. Among them the organotypic brain cultures, living slices of the CSN that can be cultured *in vitro* and challenged with bilirubin in controlled (concentration/timing) manner, strictly modelling different histopathological aspects of neurological conditions.
